# Loss of *IDH1* and *IDH2* mutations during the evolution of metastatic chondrosarcoma

**DOI:** 10.1186/s13059-025-03812-2

**Published:** 2025-11-26

**Authors:** William Cross, Iben Lyskjær, Christopher Davies, Abigail Bunkum, Ana Maia Rocha, Tom Lesluyes, Fernanda Amary, Roberto Tirabosco, Cristina Naceur-Lombardelli, PEACE consortium, Abigail Bunkum, Cristina Naceur-Lombardelli, Sonya Hessey, Kai-Keen Shiu, John Bridgewater, Daniel Hochhauser, Martin Forster, Siow-Ming Lee, Tanya Ahmad, Dionysis Papadatos-Pastos, Sam Janes, Katey Enfield, Nicholas McGranahan, Ariana Huebner, Sergio Quezada, Stephan Beck, Peter Parker, Tariq Enver, Robert E. Hynds, David R. Pearce, Mary Falzon, Ian Proctor, Ron Sinclair, Chi-wah Lok, Zoe Rhodes, David Moore, Teresa Marafioti, Miriam Mitchison, Peter Ellery, Monica Sivakumar, Mark Linch, Sebastian Brandner, Andrew Rowan, Crispin Hiley, Selvaraju Veeriah, Heather Shaw, Gert Attard, Antonia Toncheva, Paulina Prymas, Thomas B. K. Watkins, Chris Bailey, Carlos Martinez Ruiz, Kevin Litchfield, Maise Al-Bakir, Nnenna Kanu, Sophia Ward, Emilia Lim, James Reading, Benny Chain, Blanca Trujillo Alba, Tom Watkins, Melek Akay, Dhruva Biswas, Oriol Pich, Michelle Dietzen, Clare Puttick, Emma Colliver, Alistair Magness, Mihaela Angelova, James Black, Olivia Lucas, William Hill, Wing-Kin Liu, Alexander Frankell, Neil Magno, Foteini Athanasopoulou, Roberto Salgado, Claudia Lee, Kristiana Grigoriadis, Othman Al-Sawaf, Takahiro Karasaki, Imran Noorani, Sarah Benafif, Vittorio Barbe, Supreet Bola, Osvaldas Vainauskas, Anna Wingate, Daniel Wetterskog, Mahedi Hasan, Stefano Lise, GianMarco Leone, Anuradha Jayaram, Constantine Alifrangis, Ursula McGovern, Kerstin Thol, Samuel Gamble, Seng Kuong Ung, Teerapon Sahwangarrom, Claudia Peinador Marin, Sophia Wong, Piotr Pawlik, Jie Min Lam, Corentin Richard, Roberto Vendramin, Krijn Dijkstra, Jayant Rane, Jerome Nicod, Rija Zaidi, Faye Gishen, Adrian Tookman, Paddy Stone, Caroline Stirling, Samra Turajlic, James Larkin, Lisa Pickering, Andrew Furness, Kate Young, Will Drake, Kim Edmonds, Nikki Hunter, Mary Mangwende, Karla Pearce, Lauren Lewis Au, Lavinia Spain, Scott Shepherd, Haixi Yan, Ben Shum, Zayd Tippu, Brian Hanley, Charlotte Spencer, Max Emmerich, Camille Gerard, Andreas Michael Schmitt, Lyra Del Rosario, Eleanor Carlyle, Charlotte Lewis, Lucy Holt, Analyn Lucanas, Molly O’Flaherty, Steve Hazell, Hardeep Mudhar, Christina Messiou, Arash Latifoltojar, Annika Fendler, Fiona Byrne, Husayn Pallinkonda, Irene Lobon, Alex Coulton, Anne Laure Cattin, Daqi Deng, Geoffrey Hugang Feng, Andew Rowan, Nadia Yousaf, Sanjay Popat, Olivia Curtis, Charlotte Milner-Watts, Gordon Stamp, Emma Nye, Aida Murra, Justine Korteweg, Denise Kelly, Lauren Terry, Jennifer Biano, Kema Peat, Kayleigh Kelly, Peter Hill, Debra Josephs, Sheeba Irshad, James Spicer, Ula Mahadeva, Anna Green, Ruby Stewart, Natasha Wright, Georgina Pulman, Ruxandra Mitu, Sherene Phillips-Boyd, Deborah Enting, Sarah Rudman, Sharmistha Ghosh, Lena Karapagniotou, Elias Pintus, Andrew Tutt, Sarah Howlett, James Brenton, Carlos Caldas, Rebecca Fitzgerald, Merche Jimenez-Linan, Elena Provenzano, Alison Cluroe, Anna Paterson, Sarah Aitken, Kieren Allinson, Grant Stewart, Ultan McDermott, Emma Beddowes, Tim Maughan, Olaf Ansorge, Peter Campbell, Patricia Roxburgh, Sioban Fraser, Kevin Blyth, John Le Quesne, Matthew Krebs, Fiona Blackhall, Yvonne Summers, Pedro Oliveira, Ana Ortega-Franco, Caroline Dive, Fabio Gomes, Mat Carter, Jo Dransfield, Anne Thomas, Dean Fennell, Jacqui Shaw, Babu Naidu, Shobhit Baijal, Bruce Tanchel, Gerald Langman, Andrew Robinson, Martin Collard, Peter Cockcroft, Charlotte Ferris, Hollie Bancroft, Amy Kerr, Gary Middleton, Joanne Webb, Salma Kadiri, Peter Colloby, Bernard Olisemeke, Rodelaine Wilson, Ian Tomlinson, Sanjay Jogai, Samantha Holden, Tania Fernandes, Iain McNeish, Blanche Hampton, Mairead McKenzie, Allan Hackshaw, Abby Sharp, Kitty Chan, Laura Farrelly, Hayley Bridger, Rachel Leslie, Mariam Jamal-Hanjani, Charles Swanton, Simone Zaccaria, Adrienne M. Flanagan, Peter Van Loo, Mariam Jamal-Hanjani, Charles Swanton, Nischalan Pillay, Simone Zaccaria, Adrienne M. Flanagan, Peter Van Loo

**Affiliations:** 1 Rare Malignancies and Cancer Evolution Group, Centre for Cancer Research, University of Reading, Reading, UK; 2 Department of Molecular Medicine, Aarhus University Hospital, Aarhus, Denmark; 3 Department of Histopathology, Royal National Orthopaedic Hospital, Stanmore, UK; 4 Department of Pathology (Research), UCL Cancer Institute, London, UK; 5 Computational Cancer Genomics Group, UCL Cancer Institute, London, UK; 6 Cancer Metastasis Laboratory, UCL Cancer Institute, London, UK; 7 Cancer Research UK Lung Cancer Centre of Excellence, UCL Cancer Institute, London, UK; 8 Cancer Genomics Laboratory, The Francis Crick Institute, London, UK; 9 Department of Medical Oncology, University College London Hospitals, London, UK; 10 Cancer Evolution and Genome Instability Laboratory, The Francis Crick Institute, London, UK; 11 Department of Genetics, The University of Texas MD Anderson Cancer Center, Houston, TX USA; 12 Department of Genomic Medicine, The University of Texas MD Anderson Cancer Center, Houston, TX USA

**Keywords:** Chondrosarcoma, Cancer evolution, *IDH1*, *IDH2*, Metastasis, Bone tumor

## Abstract

Driver mutations in *IDH1* and *IDH2* are initiating events in the evolution of chondrosarcoma and several other cancer types. Here, we present evidence that mutant *IDH1* is recurrently lost in metastatic central chondrosarcoma. This may reflect either relaxed positive selection for the mutant *IDH1* locus, or negative selection for the hypermethylation phenotype later in tumor evolution. This finding highlights the challenge for therapeutic intervention by mutant IDH1 inhibitors in chondrosarcoma.

**Supplementary Information:**

The online version contains supplementary material available at 10.1186/s13059-025-03812-2.

## Background

Driver mutations in *IDH1* and *IDH2* have been implicated in several cancer types [1], including central chondrosarcoma [2, 3], acute myeloid leukemia, and glioblastoma. In chondrosarcoma, the evidence that *IDH1* mutations are initiating events is supported by a causative relationship with the early postzygotic conditions, Ollier disease and Maffucci syndrome [4, 5], and by mouse models [6].

There is a clear need for new treatment options for metastatic chondrosarcoma [7], as patients rarely survive beyond two years [8] and recent trials of IDH1 inhibitors report variable responses [9]. Nevertheless, emerging data suggest that *IDH1/2* mutation status has an overall impact on outcome in metastatic disease [3, 10]. To gain a deeper understanding of the role of *IDH1/2* in chondrosarcoma metastasis, we performed detailed genomic profiling of metastatic central chondrosarcoma, with a specific focus on *IDH1* and *IDH2* mutations.

## Results and discussion

**Fig. 1:**
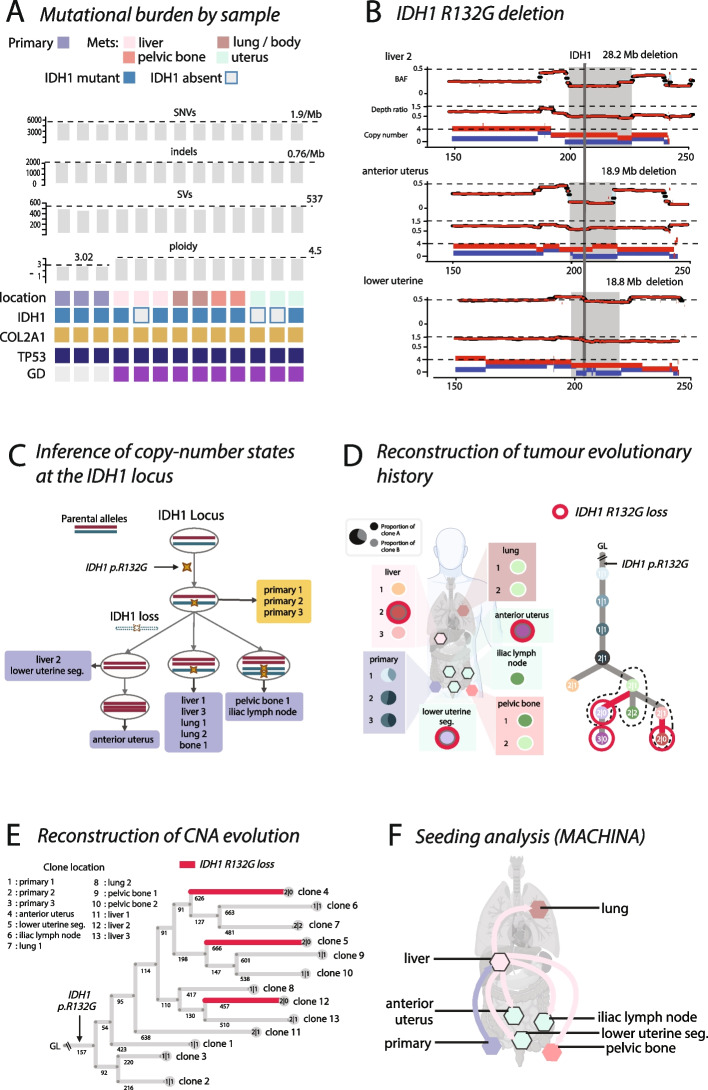
The evolution of *IDH1* mutant loss in a case of metastatic chondrosarcoma. **A** Mutational burden summary for index case CS1. **B** Driver mutation summary. The *IDH1* mutation is clonal in primary tumor samples, but absent or near absent in three metastatic sites. All metastatic sites are genome doubled. **C** Copy number states of the *IDH1* locus across sites with *IDH1* mutation loss. **D** The presence of eight clones detected are indicated in the body map. Multiple parallel losses of *IDH1* (red branches) were observed. **E** The evolutionary history of the tumor inferred via HATCHet and MEDICC2. **F** Seeding pattern determined via MACHINA suggests the liver was the first metastatic site

Our index case, CS1, is a 73-year-old patient who died of metastatic central chondrosarcoma and donated her body to research through the Cancer Research UK PEACE research autopsy study [11, 12]. We performed whole-genome sequencing on three primary tumor samples and 10 metastatic lesions to a median coverage of 120X. We called 6090 SNVs, 2718 indels, and 810 structural variants (averages: SNV: 1.9/Mb, indels: 0.76/Mb, SVs: 537/genome, Fig. 1A). The tumor’s genome was highly rearranged, had undergone whole-genome doubling, and contained driver mutations in *TP53*, *COL2A1*, and *IDH1* (Fig. 1A). While the genome doubling event was specific to metastatic tissues, we have previously reported that this alteration was not uncommon in primary high-grade chondrosarcoma [10]. No putative driver mutations specific to metastasis samples were identified. However, most strikingly, *IDH1* mutations, while present in all primary tumor samples, were absent in two metastatic samples (liver 2 and anterior uterus, both showing no mutant reads) and near-absent in a third metastatic sample (lower uterine segment, 1 mutant read, VAF = 0.01). These losses of *IDH1* mutations were associated with three different copy number losses of the *IDH1* locus on 2q (liver 2: 28.2 Mb, anterior uterus: 18.9 Mb, lower uterine segment: 18.8 Mb, Fig. 1B, Additional file 1: Table S1), suggesting the *IDH1* deletions may have occurred multiple times in parallel.

To gain deeper insight into how *IDH1* mutation losses evolved, we performed detailed subclonal reconstruction and metastatic seeding analysis, using a combination of bioinformatics tools. Subclonal reconstruction using DeCiFer [13] revealed five distinct copy number states observed at the *IDH1* locus (Fig. 1C). We combined this analysis with the phylogenetic reconstruction of SNVs using CONIPHER [14], which resulted in a single optimal phylogenetic tree, where eight distinct tumor clones were inferred and then expanded (black dotted lines) to reflect clones with different copy number states at the *IDH1* locus (Fig. 1D). This confirmed that *IDH1* mutations were lost through multiple parallel copy number losses: one in the clone present in “liver 2”, another in the clone present in the “lower uterine segment”, and another in the “anterior uterus” sample. To build further support for these results and assess the clonal relationships using an orthogonal approach, we next examined the evolution of copy number alterations by inferring clone-specific copy numbers with HATCHet [15] and reconstructing the evolutionary history of these clones using MEDICC2 [16]. This approach confirmed distinct loss of the *IDH1* locus (Fig. 1E) in each of the three samples in which *IDH1* loss was observed. To examine the seeding pattern of these clones, we used MACHINA [17], providing as input the phylogenetic tree inferred by CONIPHER for the inferred tumor clones (Fig. 1F). A single-source metastasis-to-metastasis seeding pattern was identified, suggesting that the pattern of dissemination was first to the liver, then other anatomical sites, followed by reseeding to the liver. These combined results suggest parallel evolution of *IDH1* mutation loss in this tumor.

**Fig. 2:**
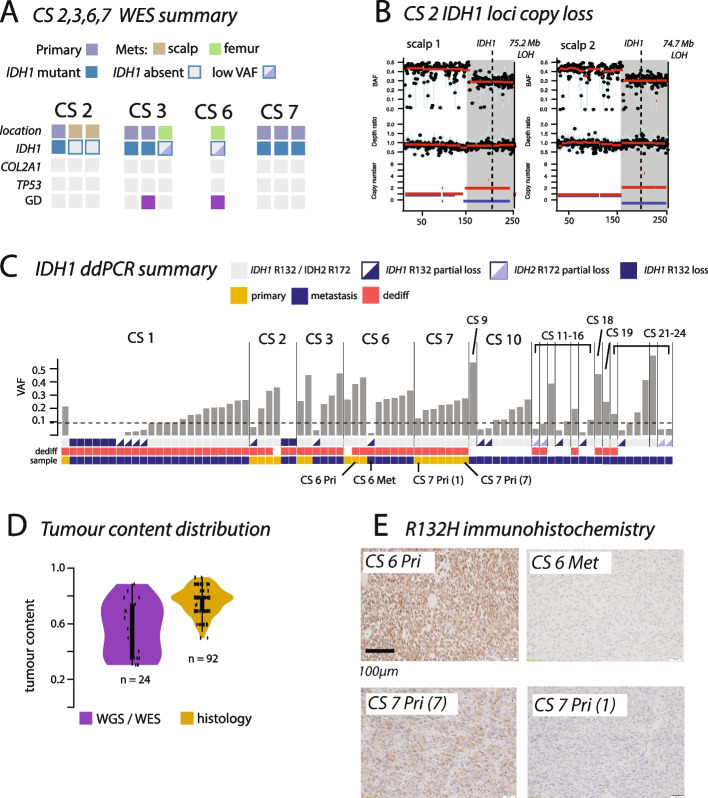
Exploration of *IDH1* and *IDH2* loss in ddPCR and immunohistochemistry. **A** Driver mutation summary of four cases subjected to exome sequencing, identified as harboring mutant *IDH1* loss. **B** Copy neutral LOH of the *IDH1* loci in CS2. **C** ddPCR frequency of *IDH1/2* mutant droplets for 19 cases of metastatic chondrosarcoma. Partial loss defined as frequencies > 1% and < 10%. Near-complete loss frequencies are defined as < 1% but higher than the background (Methods). Samples assayed for *IDH1* R132H immunostaining included in panel D are marked. **D** Tumor content distribution, as measured by sequencing and histological estimation. **E** Immunostaining for *IDH1* R132H. Loss of immunoreactivity confirmed the genetic findings in CS6_Met and CS7_Pri_1 (which had a low droplet count)

We next performed whole exome sequencing of an additional 10 tumor samples from four patients who had developed metastatic chondrosarcoma (CS2, CS3, CS6, and CS7, Fig. 2A, Additional file 1: Table S1 and Additional file 2: Table S2) to determine if *IDH1* mutation loss was a recurrent event in chondrosarcoma evolution. We identified no putative driver mutations specific to metastasis samples. We found that the primary tumor sample of patient CS2 showed a heterozygous *IDH1* R132G mutation, whereas both metastatic samples had lost this mutation due to copy neutral LOH of part of chromosome 2 (Fig. 2B). In CS3, the *IDH1* R132C mutation present in the primary tumor showed a subclonal loss in the metastasis sample (VAF = 0.073, cancer cell fraction: 21%, Fig. 2A). In patient CS6, the *IDH1* mutation was retained in the sampled metastasis (VAF = 0.23, consistent with one out of four *IDH1* copies mutated in all tumor cells). In CS7, only primary tumor samples were sequenced, and all were *IDH1* mutant. These results support our finding above that *IDH1* mutations may be recurrently lost in chondrosarcoma metastases.

To explore our findings of *IDH1* mutation losses in a larger number of samples, we used ddPCR to evaluate *IDH1* (and *IDH2*) hotspot mutation status across 78 samples from 19 patients (including 24 samples from CS1, 28 samples from CS2, CS3, CS6, CS7, and 26 samples from 14 other patients, Fig. 2C, Additional file 2: Table S3). Four out of the 19 cases had *IDH2* R172 mutations, and the remaining 15 harbored *IDH1* R132 mutations. Our results confirmed loss or near-complete loss in six metastasis samples from CS1 and two samples from CS2. We defined near-complete loss as a mutant droplet percentage < 1% but above the level of the background false positive noise of the assay (see the “Methods” section, Additional file 2: Table S3). While no additional cases showed a mutant droplet percentage < 1%, nine other cases, including three *IDH2* cases, showed mutant droplet frequencies < 10% (henceforth termed partial loss). Together, these *IDH1/2* (partial) loss events were found in 24/78 (31%) samples in 11/19 (58%) cases. These events were strongly enriched in metastasis samples compared to the primary tumor samples (24/62 *vs.* 1/16, *p* = 0.009).

The significance of partial loss events of *IDH1/2* mutations remains unclear. To formally exclude low tumor content as a possible explanation, we next evaluated tumor purity using both copy number inference from WGS/WES data and histological assessment. All samples contained a high percentage of tumor cells (mean purity from WGS/WES: 56%; mean purity from histological assessment: 74%, Fig. 2D, Additional File 2). Based on this, we hypothesize that some of these *IDH1/2* partial loss events represent subclonal mutation losses in chondrosarcoma metastases.

To provide support for the *IDH1* mutation loss detected by ddPCR at a protein level, we performed immunohistochemistry using an IDH1 R132H mutation-specific antibody on the relevant tumors with this specific alteration in primary tumor and metastasis samples from cases CS6 and CS7. CS6 showed IDH1 immunoreactivity in the primary tumor but not in the metastasis sample (Fig. 2E). The low number of *IDH1* mutant molecules detected by ddPCR in the corresponding samples (marked on Fig. 2C) likely reflects the sensitivity of the assay in detecting a minor population of mutant cells [18]. CS7 also revealed the absence of *IDH1* immunoreactivity in a single region from the primary tumor (Fig. 2E). The ddPCR result from the same region revealed a low *IDH1* R132H droplet count (13%, Fig. 1C, Additional File 2) compared to the other regions analyzed. We hypothesize that the absence of mutant *IDH1* expression may in some cases result from a combination of allelic imbalance between normal and mutant copies, and possibly clonal mixing within the sample.

In summary, by exploring the clonality of *IDH1* mutations in metastatic chondrosarcoma, we found that, despite the initiating role of *IDH1* and *IDH2* in this disease, these mutations can be recurrently lost later in tumor evolution. We note that this phenomenon has been previously observed in glioma [19] where *IDH1/2* driver mutations are also commonly observed. We hypothesize that *IDH1* and *IDH2* mutations, known to block differentiation [6], become disadvantageous later in chondrosarcoma evolution and may be subject to negative selection. Alternatively, selection for IDH1/2 mutations may become relaxed later in chondrosarcoma evolution, and losses of mutant IDH1/2 may occur as a product of mutational drift in these chromosomally instable genomes. Both models could explain the relatively high frequency of *IDH1* or *IDH2* mutation loss or partial loss in our cohort and the observation of parallel evolution for losses across multiple samples within CS1.

## Conclusions

Our study posits that initiating driver mutations in *IDH1* or *IDH2* in cartilaginous tumors are not required for the persistence of chondrosarcoma. These data support emerging evidence that *IDH1* inhibition does not consistently control disease in metastatic chondrosarcoma [20].

## Methods

### Patients and samples

We obtained archived tumor samples from 19 patients with metastatic central chondrosarcoma and *IDH1/2* mutations from the Royal National Orthopaedic Hospital. Samples from multiple tumor sites (mean 4) were available for analysis from nine of these patients, targeted digital droplet PCR (ddPCR, *n* = 19), whole exome sequencing (WES, *n* = 4), histological analysis, or a combination thereof (Additional file 1: Table S1 and Additional file 2: Table S2). In addition, fresh frozen pre- (*n* = 3) and post-mortem (*n* = 10) samples from a patient enrolled in the PEACE (Posthumous Evaluation of Advanced Cancer Environment) autopsy study were analyzed using whole genome sequencing (WGS) and histological analysis (CS1, Additional File 1 and 2). Metastatic disease was widespread and present in the liver, lung, nerve, bone, uterus, and ilium.

### Histology and immunohistochemistry

Four-micrometer sections were cut from formalin-fixed paraffin-embedded (FFPE) tissue blocks and histology assessed by AMF, RT, and FA. Immunohistochemistry was performed on cases in which an *IDH1* R132H mutation was detected on genotyping using the anti-IDH1 R132H (H09) antibody (Dianova; Hamburg, Germany; DIA-H09) on the Leica bond platform (1:100, 20 min ER1). All DNA from FFPE sources were UDG-treated prior to the IDH1 R132 assay to remove false positives caused by the deamination of nucleotides from the formalin fixation process.

### DNA extraction

5 µm diameter punches (Integra Miltex; NJ, USA) were obtained from FFPE tumor and matched normal tissue blocks from each patient. DNA was extracted from FFPE tissue using the truXTRAC FFPE total NA Plus Kit (Covaris; MA, USA; 520,252) and quantified using Nanodrop (Thermofisher; MA, USA), Qubit (Invitrogen; MA, USA; Q32851), and Tapestation (Agilent; CA, USA). DNA was extracted from frozen tumor samples and blood as recommended by manufacturers.

### Genotyping and bioinformatic analysis

We performed ddPCR for the *IDH1* R132 and *IDH2* R172 mutations as described previously [10]. In brief, we utilized non-template and negative controls to determine the background noise of the assay. This was less than 1/10,000 generated droplets, which corresponds to 0.01%. Samples with a minimum number of 10,000 droplets were included in the study, and samples with a minimum of 100 droplets with mutations were considered to harbor the mutation, as previously described [21]. We considered a droplet percentage of < 1% but above the background level to represent a near-complete loss of *IDH1* mutations, while a percentage between 1 and 10% represented a partial loss, meaning a lowering of the expected variant allele frequency. WES was performed using the Twist Exome library preparation kit, followed by paired-end sequencing on the Illumina NovaSeq 6000 platform, obtaining an average depth of 250 × (50 × for matched normal samples). WGS was performed using the TruSeq DNA PCR-Free library construction and the Illumina NovaSeq 6000 platform, using 150 bp paired-end sequencing and 100 × sequencing depth. Both the library preparation and sequencing for the WES and WGS were conducted by Macrogen (Seoul, South Korea).

Single nucleotide variants (SNVs) and indels were called on both WES and WGS data via Mutect2 (4.1.2.0), following GATK best practices. Somatic copy number alterations were called using Sequenza [22] (WES). Structural variants (SVs) were called using GRIDSS [23]. Visual inspections of driver alterations were performed using the Integrative Genomics Viewer (IGV).

### Phylogenetic analysis

We performed evolutionary and metastatic dissemination analysis of the CS1 tumor by applying a collection of existing methods to the bulk whole-genome sequencing data of multiple samples from primary and metastatic sites. Through this analysis, we aimed to (1) reconstruct the tumor phylogeny and investigate the presence of mutation losses, (2) reconstruct the evolution of copy number alterations to orthogonally assess whether parallel losses occurred at the *IDH1* locus, and (3) infer the metastatic migration patterns.

Firstly, we reconstruct the tumor evolution of distinct tumor clones using the CONIPHER algorithm [14]. Specifically, CONIPHER was executed with default parameters. We have also applied the DeCiFer algorithm [13] to investigate the possible presence of mutation losses at the IDH1 locus. DeCiFer was run on the inferred single-nucleotide variant (SNV) data excluding low-confidence SNVs such as SNVs with no copy-number information at the variant location, low variant allele frequency (< 0.2) in all samples, or low sequencing depth (< 30 reads) in any sample. Moreover, the called CNAs were provided as input to DeCiFer. DeCiFer was run using default parameters and generating state trees for mutations with maximum allele-specific copy number up to 3, maximum total copy number up to 5, and with at most 22 mutation clusters.

Secondly, we reconstructed the evolution of the inferred clone-specific CNAs using the MEDICC2 algorithm [16] with default parameters. We obtained the clone-specific input for MEDICC2 by using the HATCHet algorithm [15] to infer clone copy number profiles for each of the tumor samples. HATCHet was run on each sample individually due to the presence of different WGDs in different samples (a feature not supported by HATCHet when executed in multi-sample mode). HATCHet was run with a value of 50 genomic bin clusters, a minimum clone proportion threshold of 0.2, a maximum diploid copy number value of 12, and with the possible number of identified clones between 2 and 8 (including a diploid, normal clone).

Lastly, we reconstructed the metastatic migrations for tumor CS1 by applying the MACHINA algorithm [17] to the inferred phylogenetic tree from CONIPHER. We applied MACHINA using the default polytomy resolution mode and allowing all possible seeding patterns of increasing complexity: primary seeding only, single-source metastasis-to-metastases seeding, multi-source metastasis-to-metastases seeding, and reseeding of the primary tumor. The most parsimonious solution was chosen, corresponding to a single-source metastasis-to-metastasis seeding pattern being inferred.

### Statistical analysis

Statistical comparisons were performed using Wilcoxon tests and Fisher exact tests in the R programming language. For the allelic dropout statistics, we used binomial statistics and reported values of sequencing depth of each sample (range: 61–93X). Cancer cell fractions (CCF) of mutations were inferred from VAFs, accounting for tumor purity and copy number status, as previously described [24].

## Supplementary Information

Additional file 1. Containing CS1 summary and segmentation tables.

Additional file 2. Containing IDH1 mutations status summary and ddPCR results.

Additional file 3. Containing a list of members of the PEACE consortium.

## Data Availability

The raw genomic data was submitted under an anonymised ID within the BioProject SRA database [25]. All IDH1 mutation calls and ddPCR results are available in Additional files 1 & 2 [26].
