# Supplementary material for: Loss of IDH1 and IDH2 mutations during the evolution of metastatic chondrosarcoma
Source: Genome Biol. 2025 Nov 26;26:404. doi: 10.1186/s13059-025-03812-2 (PMC12659308; doi:10.1186/s13059-025-03812-2)
Supplement: Supplementary file 3 — Additional file 3. Containing a list of members of the PEACE consortium. [file 13059_2025_3812_MOESM3_ESM.docx]

**Members of the PEACE consortium**

Charles Swanton, Mariam Jamal-Hanjani, Simone Zaccaria, Sonya Hessey, Kai-Keen Shiu, John Bridgewater, Daniel Hochhauser, Martin Forster, Siow-Ming Lee, Tanya Ahmad, Dionysis Papadatos-Pastos, Sam Janes, Peter Van Loo, Katey Enfield, Nicholas McGranahan, Ariana Huebner, Sergio Quezada, Stephan Beck, Peter Parker, Tariq Enver, Robert E. Hynds, David R. Pearce, Mary Falzon, Ian Proctor, Ron Sinclair, Chi-wah Lok, Zoe Rhodes, David Moore, Teresa Marafioti, Miriam Mitchison, Peter Ellery, Monica Sivakumar, Mark Linch, Sebastian Brandner, Andrew Rowan, Crispin Hiley, Selvaraju Veeriah, Heather Shaw, Gert Attard, Cristina Naceur-Lombardelli, Antonia Toncheva, Paulina Prymas, Thomas B. K. Watkins, Chris Bailey, Carlos Martinez Ruiz, Kevin Litchfield, Maise Al-Bakir, Nnenna Kanu, Sophia Ward, Emilia Lim, James Reading, Benny Chain, Blanca Trujillo Alba, Tom Watkins, Melek Akay, Adrienne M Flanagan, Dhruva Biswas, Oriol Pich, Michelle Dietzen, Clare Puttick, Emma Colliver, Alistair Magness, Mihaela Angelova, James Black, Olivia Lucas, William Hill, Wing-Kin Liu, Alexander Frankell, Neil Magno, Foteini Athanasopoulou, Roberto Salgado, Claudia Lee, Kristiana Grigoriadis, Othman Al-Sawaf, Takahiro Karasaki, Abigail Bunkum, Imran Noorani, Sarah Benafif, Vittorio Barbe, Supreet Bola, Osvaldas Vainauskas, Anna Wingate, Daniel Wetterskog, Mahedi Hasan, Stefano Lise, GianMarco Leone, Anuradha Jayaram, Constantine Alifrangis, Ursula McGovern, Kerstin Thol, Samuel Gamble, Seng Kuong Ung, Teerapon Sahwangarrom, Claudia Peinador Marin, Sophia Wong, Piotr Pawlik, Jie Min Lam, Corentin Richard, Roberto Vendramin, Krijn Dijkstra, Jayant Rane, Jerome Nicod, Rija Zaidi, Faye Gishen, Adrian Tookman, Paddy Stone, Caroline Stirling, Samra Turajlic, James Larkin, Lisa Pickering, Andrew Furness, Kate Young, Will Drake, Kim Edmonds, Nikki Hunter, Mary Mangwende, Karla Pearce, Lauren, Lewis Au, Lavinia Spain, Scott Shepherd, Haixi Yan, Ben Shum, Zayd Tippu, Brian Hanley, Charlotte Spencer, Max Emmerich, Camille Gerard, Andreas Michael Schmitt, Lyra Del Rosario, Eleanor Carlyle, Charlotte Lewis, Lucy Holt, Analyn Lucanas, Molly O'Flaherty, Steve Hazell, Hardeep Mudhar, Christina Messiou, Arash Latifoltojar, Annika Fendler, Fiona Byrne, Husayn Pallinkonda, Irene Lobon, Alex Coulton, Anne Laure Cattin, Daqi Deng, Geoffrey Hugang Feng, Andew Rowan, Nadia Yousaf, Sanjay Popat, Olivia Curtis, Charlotte Milner-Watts, Gordon Stamp, Antonia Toncheva, Emma Nye, Aida Murra, Justine Korteweg, Denise Kelly, Lauren Terry, Jennifer Biano, Kema Peat, Kayleigh Kelly, Peter Hill, Debra Josephs, Sheeba Irshad, James Spicer, Ula Mahadeva, Anna Green, Ruby Stewart, Natasha Wright, Georgina Pulman, Ruxandra Mitu, Sherene Phillips-Boyd, Deborah Enting, Sarah Rudman, Sharmistha Ghosh, Lena Karapagniotou, Elias Pintus, Andrew Tutt, Sarah Howlett, James Brenton, Carlos Caldas, Rebecca Fitzgerald, Merche Jimenez-Linan, Elena Provenzano, Alison Cluroe, Anna Paterson, Sarah Aitken, Kieren Allinson, Grant Stewart, Ultan McDermott, Emma Beddowes, Tim Maughan, Olaf Ansorge, Peter Campbell, Patricia Roxburgh, Sioban Fraser, Kevin Blyth, John Le Quesne, Matthew Krebs, Fiona Blackhall, Yvonne Summers, Pedro Oliveira, Ana Ortega-Franco, Caroline Dive, Fabio Gomes, Mat Carter, Jo Dransfield, Anne Thomas, Dean Fennell, Jacqui Shaw, Babu Naidu, Shobhit Baijal, Bruce Tanchel, Gerald Langman, Andrew Robinson, Martin Collard, Peter Cockcroft, Charlotte Ferris, Hollie Bancroft, Amy Kerr, Gary Middleton, Joanne Webb, Salma Kadiri, Peter Colloby, Bernard Olisemeke, Rodelaine Wilson, Ian Tomlinson, Sanjay Jogai, Samantha Holden, Tania Fernandes, Iain McNeish, Peter Ellery, Blanche Hampton, Mairead McKenzie, Allan Hackshaw, Abby Sharp, Kitty Chan, Laura Farrelly, Hayley Bridger, Rachel Leslie.
